# Characterizing Hospitalization Cost for Term Newborns in Intensive Care: A Multi-Center Analysis Across U.S. Children’s Hospitals

**DOI:** 10.21203/rs.3.rs-10592460/v1

**Published:** 2026-08-10

**Authors:** Christhian Cano Guerra, Matt Hall, Jay Berry, John Zupancic, Brian King

**Affiliations:** Boston Children’s Hospital; Harvard Medical School; Beth Israel Deaconess Medical Center

## Abstract

**Objective:**

To characterize and compare direct medical costs, cost drivers, and diagnoses among term newborns admitted to neonatal and non-neonatal ICUs at U.S. children’s hospitals.

**Study Design:**

Retrospective cohort study of term newborns (≥ 37 weeks’ gestation) admitted to ICUs within 7 days of birth between 1/1/2022 and 12/31/2024, using the Pediatric Health Information System.

**Results:**

Among 45,738 term infants at 42 hospitals, non-neonatal ICU admissions represented 8% of encounters but 25% of costs ($912 million). Median cost was $20,367 [IQR: $8,336 - $59,887], with room and board as the primary driver in both settings. Higher costs were independently associated with medical complexity (≥ 4 chronic conditions; RR 6.05, 95% CI 5.15–7.09) and illness severity (RR 1.07 per unit increase, 95% CI 1.07–1.07).

**Conclusions:**

Term newborns requiring intensive care incurred high costs, driven by medical complexity and acuity, providing national benchmark data to inform future efforts to optimize care delivery.

## Introduction

Hospitalizations of newborns and infants account for nearly three-quarters of pediatric admissions in the United States.(1) While traditionally centered on preterm infants, neonatal intensive care units (NICUs) are now admitting a growing number of term and normal-weight newborns.(2–4) Research indicates that more than 40% of infants admitted to the highest-level NICUs (Level IV)(5) are full-term, representing nearly one-fifth of total hospital days and close to a quarter of total NICU costs, with fewer than half of these admissions for high-acuity cases.(6, 7) This trend has raised concerns about possible overuse of high-acuity neonatal care.(3, 8, 9) However, the clinical drivers and economic implications of high-level care among term newborns remain incompletely understood.

Existing cost estimates for term newborns are not representative of those receiving care in the highest acuity environments, such as Level IV NICUs or pediatric ICUs within children’s hospitals. (5, 10) Estimated costs for term newborns are typically derived from regional or population-based cohorts that include large numbers of healthy infants and do not account for the level or intensity of the care provided.(11, 12) The mean cost of an uncomplicated term birth hospitalization has been estimated at approximately $2,500, while national, multi-center studies of infants admitted to Level III and IV NICUs report substantially higher costs.(7, 12) As a result, the true costs associated with intensive care utilization among term newborns, as well as the patterns of resource utilization in this population, remain poorly characterized.

Unlike preterm infants, whose care is almost exclusively provided in NICUs, term newborns could require admission to other care environments within children’s hospitals, including pediatric or cardiac ICUs, reflecting the role of these institutions as referral centers for complex neonatal conditions. These care pathways likely differ substantially in their clinical profiles, resource intensity, and cost structures. Although the available literature is explicit about differences in hospital costs among NICUs and other ICUs for those undergoing congenital heart surgery, these studies did not characterize the broader term population, their economic burden, and diagnostic profiles.(13, 14) Understanding these differences is important to optimize resource allocation, staffing models, and value-based care strategies at children’s hospitals.

To the best of our knowledge, no national, multi-center study has directly compared the diagnosis profiles and economic burden of term newborn admissions across neonatal and non-neonatal ICUs. To address these gaps, we conducted a national retrospective cohort study to estimate ICU costs and their drivers among term newborns. Our specific aims were (1) to identify the most common diagnoses associated with admission to neonatal and non-neonatal ICUs among term newborns, (2) to quantify direct medical costs, characterize principal cost drivers, and compare costs and resource utilization between ICU settings, and (3) to determine the association of infants’ demographic and clinical characteristics with direct hospitalization costs. By describing patterns in diagnoses and costs among term newborns receiving high-acuity care, this study aims to provide foundational evidence to inform future investigations of value-based neonatal care delivery.

## Methods

### Study design

We conducted a retrospective cohort study of term newborns admitted to or transferred to ICUs at children’s hospitals across the United States, whose data were collected by the Pediatric Health Information System (PHIS). The study was reviewed by the Boston Children’s Hospital Institutional Review Board and determined to be exempt because it used de-identified personal health information and administrative data.

### Data source

PHIS is an administrative database that includes hospitalization data from more than 47 children’s hospitals that are part of the Children’s Hospital Association (Lenexa, KS). The database includes demographic information, diagnoses, procedural codes (using the *International Classification of Diseases, Tenth Revision, Clinical Modifi*cation [ICD-10-CM]), and daily resource utilization and costs. Resources at each hospital are mapped to a standard set of clinical transaction codes (CTC), organized into imaging studies, clinical services, laboratory tests, pharmacy, supplies, and room and boarding charges, enabling comparisons across institutions. Data undergo reliability and validity checks and must meet a specific quality threshold before being incorporated.

### Cohort Identification

We identified term newborns (gestational age [GA] ≥ 37 weeks) admitted or transferred after birth to ICUs at PHIS-participating children’s hospitals between January 1st, 2022, and December 31st, 2024. To maximize cohort accuracy and reduce misclassification, our exclusion criteria were designed to avoid including infants admitted to the hospital after discharge home and for whom descriptive data could misclassify them as term newborns (i.e., premature infants with corrected age > 37 weeks). As a result, we excluded infants with ICD-10-CM codes that categorize them as premature at birth (P07 series), as described in Supplemental Table 1. We predefined cutoffs for discrete variables and excluded observations with birth weight ≥ 6,000 g and GA at birth ≥ 45 weeks, which were suggestive of data quality issues or errors. Those admitted to the hospital via the Emergency Department (ED) or to other settings rather than the ICU, and those transferred or admitted after 7 days of life, were excluded. The latter accounts for the need to include those requiring non-urgent transfer after the birth admission, while still likely being admitted for diagnoses associated with perinatal and/or congenital conditions requiring specialized care.

### Main Outcome Measure

#### Main Outcome Measure

The PHIS database captures detailed daily hospital charges from each participating hospital. Charges are submitted for each item used (e.g., laboratory, imaging, etc.) and then converted to costs using annual hospital- and department-level cost-to-charge ratios (CCR). Using the Center for Medicaid & Medicaid Services wage and price index, these estimates are also normalized to account for geographic differences in labor and other input costs.(15) This ensures that cost-variation assessments reflect only clinical resource utilization across participating hospitals, rather than pricing structures or regional cost environments. All costs are hospital-reported direct medical costs and exclude physician billing and out-of-pocket costs incurred by families.

Costs were calculated for each patient who met the inclusion criteria, including the total hospitalization costs across both ICU and non-ICU settings, to comprehensively assess total admission costs as a measure of resource utilization. Cost values are reported in nominal U.S. dollars ($), reflecting the year of service, spanning the 2022–2024 study period, without adjustment for inflation.

### Demographic and Clinical Characteristics

Demographic information included sex, GA (divided by completed weeks at birth from 37 to > = 42 weeks), birth weight (classified as < 2,000 g, >= 2,000 to < 4,000 g, and > 4,000), race/ethnicity (categorized as Non-Hispanic White, Non-Hispanic Black, Hispanic, Asian or Pacific Islander, and other), age at admission at days of life (DOL) 0, 1, 2, and > = 3 days, source of admission (outborn and inborn), discharge disposition (Home/Home health, skilled facility, death, and other), and primary payer (government, commercial, and other). Each patient was assigned a Child Opportunity Level (very low, low, moderate, high, or very high) based on a composite index of features that support children’s thriving and reflect neighborhood opportunities in education, health, and socioeconomic outcomes, derived from their residential ZIP code.(16)

Patients were classified into two categories based on the first ICU required after admission: the NICU or non-neonatal ICUs (Pediatric or Cardiac ICUs). The admitting unit was designated as the primary exposure variable for between-group comparisons. Length of stay (in days) and the need for surgical procedures (yes/no) were determined and stratified by admitting unit.

To establish diagnoses associated with ICU admissions, we use the Pediatric Clinical Classification System (PECCS). PECCS classifies ICD-10-CM codes into clinically meaningful, mutually exclusive diagnoses, with a focus on pediatric conditions and treatments.(17, 18) Each encounter is assigned a single PECCS diagnosis based on the principal diagnosis as flagged on discharge in the PHIS database. Conditions are also classified as medical, surgical, or medical/surgical.

To estimate the association with cost and medical complexity, children were also classified using the Complex Chronic Condition (CCC) ICD code grouping. The CCC code grouping identifies children using a validated algorithm and includes those with diagnoses and procedural codes associated with any medical condition expected to last at least 12 months, involving multiple organs or systems, and requiring pediatric subspecialty care.(19, 20) Children were assigned to 1 of 4 categories depending on the number of CCCs (0, 1, 2, 3, >=4) associated with the evaluated encounter. Children could be assigned to more than one CCC category during a single hospitalization.

Acuity was determined using the Hospitalization Resource Intensity Score for Kids (H-RISK), a validated APR-DRG–based measure of expected pediatric resource utilization available within PHIS. H-RISK is expressed as a relative weight ratio based on the deviation in cost for each APR-DRG and severity level from the overall mean cost of all patients in a national pediatric hospitalization database. A higher score indicates greater severity and resource utilization.(21)

### Statistical Analysis

Categorical variables were compared between NICU and non-neonatal ICU hospitalizations using chi-square tests. Given the large sample size, standardized mean differences (SMDs) were calculated to assess imbalance regardless of sample size. For binary and multi-level categorical variables, SMDs were calculated as the difference in group proportions divided by the pooled standard deviation of the proportion. For variables with more than two categories, the maximum absolute SMD across all category levels was reported. Consistent with established conventions, an absolute SMD < 0.10 was considered a negligible imbalance; 0.10–0.20 small; 0.20–0.50 moderate; and > 0.50 large imbalance.

We determined the overall cohort cost and the proportion of costs attributed to the NICU and non-neonatal ICUs. At the patient level, costs were summarized using medians and interquartile ranges (IQRs) due to skewed distributions. Median per-patient per-day costs were also calculated. Costs were stratified by admission unit, resource type, and by day of life at admission. Resource use was summarized using median percentages of the total cost attributed to each category. At the population level, diagnosis-specific costs were calculated by aggregating costs across hospitalizations within each PECCS category. We used generalized estimating equations (GEE) with a gamma distribution and log link to model hospitalization costs, accounting for within-hospital clustering to identify demographic and clinical characteristics independently associated with higher costs. Covariates included demographics (sex, GA, birth weight, and race), acuity (H-RISK), medical complexity (CCC), and admission setting.

Diagnoses were classified by ICU setting, ranked by frequency, and by cost contribution relative to the total cost. We then identified the most prevalent and most costly clinical diagnoses among infants admitted to the NICU and non-neonatal ICU, using rankings based on frequency and cost proportion. The proportion of total cost attributable to each diagnosis was calculated by dividing the cost for each diagnosis by the total cost for that admission setting. Pareto charts were constructed to identify the subset of diagnoses accounting for 80% of total costs in each intensive care setting. All statistical analyses were conducted using SAS v.9.4 (SAS Institute, Cary, NC), and p < 0.05 was considered statistically significant. Figures were rendered using Python’s Matplotlib library (version 3.10).(22)

## Results

Between January 1, 2022, and December 31, 2024, a total of 280,209 term newborn encounters were identified. Of the 47 children’s hospitals that contributed data from the NICU and non-neonatal ICU during the study period, five were excluded due to incomplete reporting of certain variables: four institutions did not report GA, and one institution did not report birth weightAfter applying exclusion criteria, a total of 45,738 encounters across 42 hospitals were admitted to ICUs and included in the final cohort. A flow diagram describing our inclusion and exclusion criteria is included in the supplemental material (Supplemental Fig. 1).

### Subject Characteristics

Cohort characteristics, stratified by ICU admission setting, are summarized in Table 1. Of the 45,738 hospitalizations included during the study period, 42,003 (91.8%) were initially admitted to the NICU and 3,735 (8.2%) to non-neonatal ICUs. Children were predominantly male (57.2%), non-Hispanic white (45.9%), born at 39 weeks’ gestation (32.2%), and admitted on the day of birth (67.5%). The most common BW category was 2,000–4,000 grams (88.7%). Medical complexity, defined by ≥ 1 CCC, was present in 43.8% of infants. Surgical procedures were performed in 15.2% of hospitalizations. Compared to infants admitted to NICUs, those in the non-neonatal ICUs had higher rates of surgical intervention (56.1% vs 11.6%).

Compared with non-neonatal ICU hospitalizations, NICU admissions varied significantly across nearly all demographic and clinical characteristics (all p-values < 0.001 except for sex). However, the differences were small in magnitude for sex, race, and age at admission (all SMD < 0.2), whereas differences were largest for surgical procedures (SMD 1.20) and chronic condition burden (0.84), and more modest for source of admission (0.34), length of stay (0.43), and discharge disposition (0.28).

### Direct Medical Costs

Total cohort costs during the study period were $3.7 billion. Among those classified as non-neonatal ICU admissions (~ 8%), the total cost was $912 million, accounting for ~ 25% of the cohort’s total costs. Costs differed substantially between ICU admission settings, consistent with the differences in clinical complexity and surgical burden described above. The median hospitalization cost was $20,367 [IQR $8,336, $59,887], with those admitted to the non-neonatal ICU incurring higher total ($115,608 vs $18,362) and per-day costs ($6,796 vs $4,293) than those in the NICU (Table 2). Room and board costs accounted for the largest share of costs in both ICU settings (Fig. 1).

Median hospitalization costs varied substantially by day of life at admission (Supplemental Table 2). In the entire cohort, infants admitted on DOL 1 and 2 had the highest median costs ($28,411 and $29,151, respectively), surpassing both birth admissions (DOL 0: $18,771) and later admissions (day ≥ 3: $14,452). This trend persisted in the NICU subgroup, where admissions on DOL 1–2 were associated with median costs approximately 50–60% higher than those on DOL ≥ 3. Conversely, in the non-neonatal ICU cohort, the highest median costs occurred among infants admitted on DOL 0 ($128,885), with median costs decreasing for later admissions (DOL 1: $98,656 vs DOL ≥ 3: $ 79,113).

In the adjusted GEE model (Supplemental Table 3), CCCs were strongly associated with higher costs, which increased more than sixfold among infants with ≥ 4 chronic conditions (RR 6.05, 95% CI 5.15–7.09; p < 0.001). Higher severity (as measured by the H-RISK) was also independently associated with higher cost (RR 1.07 per unit increase; 95% CI 1.07–1.07; p < 0.001). Compared with infants born at ≥ 42 weeks’ gestation, those born at 39–41 weeks had significantly lower costs. Location of care (NICU vs non-neonatal ICU), sex, and race were not independently associated with cost after adjustment.

### Diagnoses associated with ICU admission and associated costs

Figure 2 shows the distribution of costs per PECCS categories across both ICU settings. When ranked by total cost across admission settings, the top 26 most costly conditions in the NICU accounted for approximately 80% of total hospitalization costs ($2.3 billion), compared with the top 12 in the non-neonatal ICU ($740 million).

The “Liveborn” category was the most prevalent in both the NICU and the non-neonatal ICU, accounting for 15,129 hospitalizations, or 33.1% of all hospitalizations during the study. The cumulative hospitalization costs for those with this condition exceeded $967 million, representing 26.1% of the entire cohort’s costs (see Supplemental Tables 4a and 4b). Interestingly, there were notable differences in median costs by admission setting: $10,492 [IQR $5,072, $25,638] for NICU versus $168,002 [IQR $51,627, $343,064] for non-neonatal ICU.

In both ICU settings, the conditions with higher cumulative costs were more likely to be surgical (68% in the non-neonatal ICU; 50% in the NICU), followed by medical (30% in the non-neonatal ICU; 46% in the NICU), and in a small proportion, medical/surgical (2% in the non-neonatal ICU; 4% in the NICU). Several of the costliest conditions in both ICU settings accounted for a disproportionately large share of total costs relative to their prevalence (e.g., anomalies of the diaphragm: total costs exceeding $100 million, or ~ 3% of total NICU costs, but accounting for less than 1% of subjects).

## Discussion

In this retrospective national cohort study of over 45,000 term newborns requiring intensive care at 42 U.S. children’s hospitals, we found that intensive care admissions accounted for more than $3.7 billion in healthcare costs over three years, or around $1.2 billion per year. Our findings reveal that term newborns requiring intensive care generated a significant economic burden that is shaped by two markedly different clinical profiles, depending on the ICU setting.

Previous studies estimating costs for term newborns reported much lower hospitalization costs owing to the large proportion of term infants who are considered low risk and do not require intensive care. (11, 12) In contrast, median hospitalization costs in this dataset of newborns requiring intensive care can be 10 to 50 times higher, driven by increased resource use, surgical requirements, and longer hospital stays. Although NICU admissions comprised the larger proportion of admissions, non-neonatal ICU admissions accounted for a small share of encounters (8%) but nearly a quarter of the total cohort costs. The high concentration of infants with congenital heart disease explained the sixfold increase in hospitalization costs, reflecting the high procedural intensity and surgical burden of this group. (23, 24) As in studies of premature infants, room and board costs were the main drivers of hospitalization costs in both ICUs, accounting for up to 80% of total costs. (25)

These differences in cost structure across settings suggest that cost-reduction and quality improvement strategies for term newborns requiring ICU care should be tailored to the admission setting: those in non-neonatal ICUs may achieve the greatest impact by targeting high-cost congenital heart diagnoses, while NICU initiatives would benefit from a broader, system- and complexity-based approach. Compared with recently published estimates, our study found that NICU hospitalization costs continue to rise, underscoring the significant economic burden this population imposes, likely due to rising healthcare costs, greater medical complexity, and longer LOS. (7). As an example, Intrauterine Hypoxia and Asphyxia, a common cause of admission for term infants to the NICU, exceeded median costs of $67,000 per hospitalization, a slight increase from prior literature reports.(26)

Our analysis showed distinct cost patterns by day of life at admission. Infants admitted to the NICU on DOL 1 and 2 had higher median costs than those admitted at birth or later, possibly due to delayed respiratory, metabolic, or physiologic issues or transfer after initial stabilization at birth hospitals. Non-neonatal ICU admissions had the highest costs on day 0, aligning with immediate management of critical congenital anomalies found through prenatal screening or early evaluation. These patterns suggest that the timing of referral and transfer affects utilization and cost. Early or prenatal detection of anomalies enables prompt care and may improve outcomes, while concentrating high costs on the first day. Later NICU admissions may reflect evolving issues or delayed recognition, indicating opportunities for earlier risk assessment and targeted treatment. These patterns support the value of prenatal diagnosis and early transfer planning as potential levers for reducing resource burden, particularly for term infants with anticipated high-acuity needs.

Medical complexity and illness severity were the strongest independent predictors of costs, rather than demographic factors or ICU type. A clear dose-response relationship was observed between the number of CCCs and hospitalization costs, with those carrying 4 or more CCCs experiencing a 600% increase in costs compared with those without any. This finding has been extensively documented in the pediatric literature, which emphasizes that those with increasing medical complexity incur disproportionately higher costs and healthcare utilization during the initial hospitalization and beyond.(27–29) These findings reinforce that, among term infants admitted to high-level intensive care, clinical acuity and medical complexity are the principal determinants of resource utilization.

The “Liveborn” category was the most frequent PECCS category among both NICU and non-neonatal intensive care units and accounted for the highest share of hospitalization costs. This category captures individuals who were not assigned a specific perinatal diagnosis upon admission from birth hospitals or lower levels of care, or who were “automatically” assigned a code by the electronic medical record. Within the database, it is not feasible to determine the reason for intensive care use admission from the ICD code list assigned to each encounter. Moreover, the lack of a “timestamp” for each ICD code makes it even more challenging. As such, a majority of these encounters may be misclassified, given their higher-than-expected median hospitalization costs relative to prior studies of uncomplicated term newborns, a difference that is more pronounced in the non-neonatal ICU population, where median costs surpassed $168,000. The “liveborn” category was retained in the cohort to preserve representativeness and true healthcare resource utilization of the term infant population, but its heterogeneity limits the interpretability of diagnosis-specific cost findings. Future studies focusing on diagnostic cost benchmarking should account for this coding artifact.

Perinatal complications and respiratory conditions were the most frequent causes of admission to the NICU and contributed substantially to overall costs. This finding aligns with prior literature documenting that respiratory compromise and transitional physiologic instability in term infants are frequent drivers of intensive care, confirming this pattern at a national, multisite level.(30–32) Although less prevalent, those classified as surgical had the highest hospitalization costs and per-subject costs. Congenital anomalies of the diaphragm, esophageal atresia and tracheoesophageal fistula, and gastroschisis were among those exceeding median costs of $100,000 per hospitalization, which aligns with previous studies reporting hospitalization costs among pediatric surgical conditions.(33)

In contrast, admissions to non-neonatal ICUs were dominated by complex congenital heart disease and other surgical conditions. Diagnoses such as hypoplastic left heart syndrome, transposition of the great vessels, and coarctation of the aorta were among the costliest in non-neonatal settings, consistent with the literature demonstrating high resource use for neonatal cardiac surgical care.(23, 24, 34, 35) These findings are consistent with the role of children’s hospitals as referral centers for neonatal cardiac and surgical care and underscore the intensity of resource use required for these conditions.(13, 36) The presence of congenital cardiac diagnoses in both ICU settings reflects institutional differences in care, with some infants receiving initial stabilization in the NICU and then transferring to the non-neonatal ICU for postoperative care. Taken together, these diagnosis-specific patterns confirm that NICU and non-neonatal ICU admissions represent fundamentally distinct cost populations among term newborns, underscoring the importance of setting-stratified approaches to cost analysis and benchmarking in this group.

## Conclusion

Term newborns admitted to intensive care units at U.S. children’s hospitals form a clinically diverse and economically important group. Medical complexity and illness severity were the main predictors of costs, whereas demographic factors and ICU type were not independently associated with cost after adjustment. A small number of diagnoses, especially respiratory conditions and congenital anomalies, accounted for a major share of expenses. Our findings provide up-to-date national data to guide clinical, operational, and research efforts to understand resource use among high-acuity term infants.

## Limitations

This study has a few limitations inherent in its retrospective nature and reliance on administrative data. First, the data collected from PHIS are from a subset of children’s hospitals that can serve as proxies for the highest level of care (Level III and Level IV NICUs, Pediatric ICUs, Cardiac ICUs), but may limit the generalizability of our results, as they may not be representative of the national population. Second, diagnoses, medical complexity, and resource utilization were identified using ICD-10-CM diagnosis and procedure codes. Even though this is the most reliable method for ascertaining diagnoses in term newborns, it is prone to misclassification due to database limitations and variability in coding practices across institutions. Third, standardized direct medical costs reflect hospital-reported resource use but exclude professional fees, post-discharge expenditures, and societal costs, limiting comprehensive economic assessment. Additionally, it is worth noting that the cost incurred before transfer to the studied institutions could be significant, and they were not accounted for in the present analysis. Fourth, although models adjusted for severity using H-RISK and CCC classifications, residual confounding related to physiologic severity or clinical decision-making is possible. Finally, excluding infants admitted through the emergency department may limit the generalizability of our findings to those requiring the highest level of care; however, this was necessary to ensure a more homogeneous cohort of neonatal admissions due to perinatal causes, including interfacility transfers.

## Supplementary Material

Supplementary Files

This is a list of supplementary files associated with this preprint. Click to download.

- SupplementalMaterialPHISTerminfants.docx

## Figures and Tables

**Figure 1:**
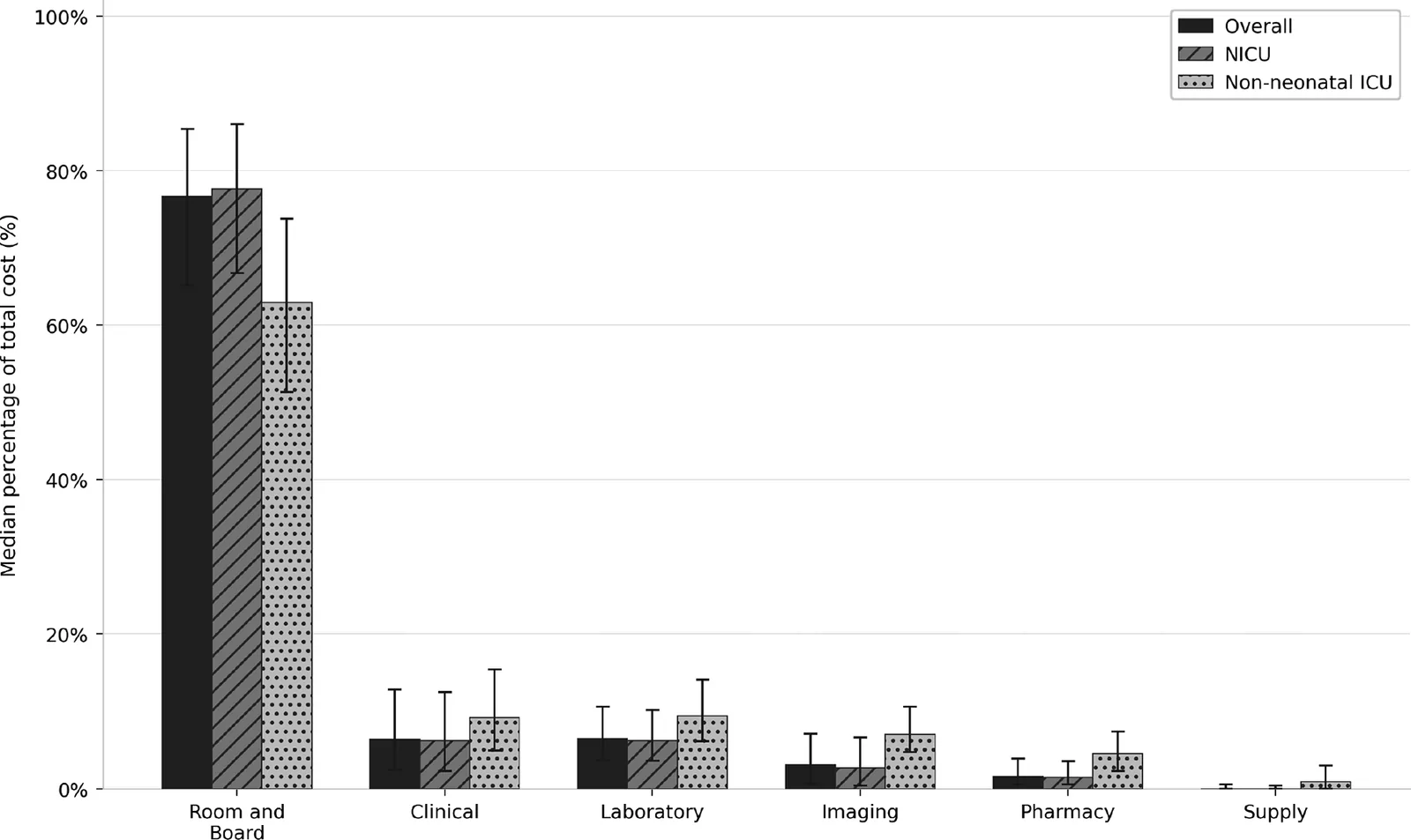
Distribution of Costs by Resource Category among Term Newborns Admitted to Intensive Care Units at U.S. Children’s Hospitals, 2022–2024. Bars represent the median percentage of total hospitalization cost per patient; error bars indicate the interquartile range (P25–P75). Categories are ordered by median cost contribution in the overall cohort.

**Figure 2:**
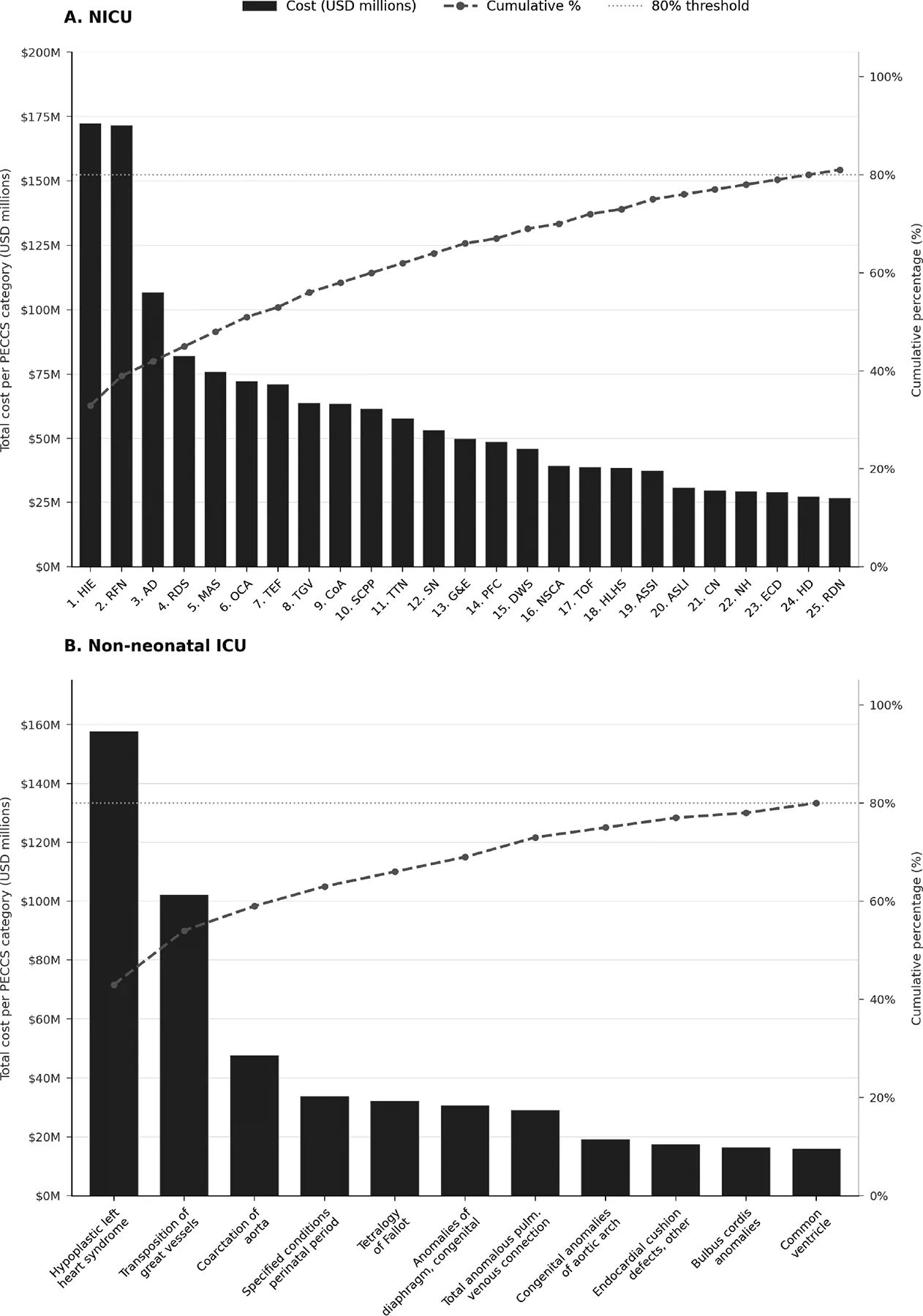
Distribution of Costs by PECCS Categories among Term Infants Admitted to the ICU at U.S. Children’s Hospitals. **Panel A.** **Neonatal ICU**: The “Liveborn” category is not shown in the cost distribution figure. In this setting, “Liveborn” accounts for approximately 26% of total NICU costs, equivalent to USD 736 million. **Panel B.** **Non-neonatal ICU**: The “Liveborn” category is also not shown, which represents approximately 25% of total non-neonatal ICU costs, totaling more than USD 239 million. Abbreviations: **HIE**: Hypoxic-ischemic encephalopathy (intrauterine hypoxia & birth asphyxia); **RFN**: Respiratory failure of newborn; **AD**: Anomalies of diaphragm, congenital; **RDS**: Respiratory distress syndrome in newborn; **MAS**: Meconium aspiration syndrome; **OCA**: Other congenital anomalies; **TEF**: Tracheoesophageal fistula / esophageal atresia & stenosis, congenital; **TGV**: Transposition of great vessels; **CoA**: Coarctation of aorta or interrupted aortic arch; **SCPP**: Specified conditions originating in perinatal period; **TTN**: Transient tachypnea of newborn; **SN**: Sepsis of newborn; **G&E**: Gastroschisis & exomphalos; **PFC**: Persistent fetal circulation; **DWS**: Drug withdrawal syndrome in newborn; **NSCA**: Nervous system congenital anomalies; **TOF**: Tetralogy of Fallot; **HLHS**: Hypoplastic left heart syndrome; **ASSI**: Atresia & stenosis of small intestine, congenital; **ASLI**: Atresia & stenosis of large intestine, rectum & anal canal, congenital; **CN**: Convulsions in newborn; NH: Neonatal hypoglycemia; **ECD**: Endocardial cushion defects, other; **HD**: Hirschsprung’s disease & other congenital functional disorders of colon; **RDN**: Respiratory distress of newborn.

**Table 1:** Demographics and Clinical Characteristics of Term Newborns Admitted to Intensive Care Units at U.S. Children’s Hospital between January 1^st,^ 2022, and 31st December 2024.

Characteristics*		Overall	NICU	Non-neonatal ICU	Max Abs SMD‡
	Total	45,738	42,003 (91.8)	3,735 (8.2)	
Sex, *N (%)*	Male	26,117 (57.2)	23,937 (57)	2,180 (58.5)	0.03 (N)
	Female	19,574 (42.8)	18,027 (43)	1,547 (41.5)	
GA (weeks), *N (%)*	37	12,502 (27.3)	11,626 (27.7)	876 (23.5)	0.22 (M)
	38	9,428 (20.6)	8,551 (20.4)	877 (23.5)	
	39	14,731 (32.2)	13,179 (31.4)	1,552 (41.6)	
	40	6,638 (14.5)	6,283 (15)	355 (9.5)	
	41	2,251 (4.9)	2,181 (5.2)	70 (1.9)	
	>=42	188 (0.4)	183 (0.4)	5 (0.1)	
BW (grams), *N (%)*	< 2,000	664 (1.5)	625 (1.5)	39 (1)	0.13 (S)
	>=2,000 - <4,000	40,551 (88.7)	37,100 (88.3)	3,451 (92.4)	
	>=4,000	4,523 (9.9)	4,278 (10.2)	245 (6.6)	
Race and Ethnicity, *N (%)*	Non-Hispanic White	20,977 (45.9)	19,262 (45.9)	1,715 (45.9)	0.07 (N)
	Non-Hispanic Black	6,579 (14.4)	6,122 (14.6)	457 (12.2)	
	Hispanic	8,762 (19.2)	8,024 (19.1)	738 (19.8)	
	Asian or Pacific islander	2,017 (4.4)	1,854 (4.4)	163 (4.4)	
	Other	7,403 (16.2)	6,741 (16)	662 (17.7)	
Age on admission (days), *N (%)*	0	30,881 (67.5)	28,266 (67.3)	2,615 (70)	0.06 (N)
	1	7,320 (16)	6,778 (16.1)	542 (14.5)	
	2	3,002 (6.6)	2,741 (6.5)	261 (7)	
	>=3	4,535 (9.9)	4,218 (10)	317 (8.5)	
Source of Admission, *N (%)*	Outborn	30,535 (66.8)	27,501 (65.5)	3,034 (81.2)	0.34 (M)
	Inborn	15,203 (33.2)	14,502 (34.5)	701 (18.8)	
Surgical procedure, *N (%)*	Yes	6,965 (15.2)	4,868 (11.6)	2,097 (56.1)	1.20 (L)
CCCs	0	25,696 (56.2)	25,027 (59.6)	669 (17.9)	0.84 (L)
	1	12,332 (27)	10,784 (25.7)	1,548 (41.4)	
	2	4,243 (9.3)	3407 (8.1)	836 (22.4)	
	3	1,839 (4)	1480 (3.5)	359 (9.6)	
	>=4	1,628 (3.6)	1305 (3.1)	323 (8.6)	
COI, *N (%)*	Very Low	1,952 (17)	1705 (17.2)	247 (15.8)	0.11 (S)
	Low	2,491 (21.7)	2203 (22.2)	288 (18.4)	
	Moderate	2,568 (22.4)	2225 (22.4)	343 (21.9)	
	High	2,318 (20.2)	1942 (19.6)	376 (24)	
	Very High	2,157 (18.8)	1,844 (18.6)	313 (20)	
Disposition, *N (%)*	Home/Home Health	41,313 (90.3)	38,112 (90.7)	3,201 (85.7)	0.28 (M)
	Skilled Facility	2,724 (6)	2,502 (6)	222 (5.9)	
	Death	805 (1.8)	613 (1.5)	192 (5.1)	
	Other	896 (2)	776 (1.8)	120 (3.2)	
Payer, *N (%)*	Government	21,951 (48)	20,258 (48.2)	1,693 (45.3)	0.12 (S)
	Commercial	21,013 (45.9)	19,099 (45.5)	1,914 (51.2)	
	Other	2,774 (6.1)	2,646 (6.3)	128 (3.4)	
LOS, Median *[IQR]*	Days	5 [2, 12]	5 [2, 11]	15 [5, 30]	0.43 (M)

* p-values were calculated using χ^2^ tests for categorical variables. All values were p < 0.001, except for sex.

‡ Maximum absolute SMD: For multi-category variables, the **maximum absolute SMD across category levels** is reported. An SMD < 0.10 was considered a negligible imbalance (N); 0.10–0.20 small (S); 0.20–0.50 moderate (M); >0.50 large imbalance (L).

Abbreviation: **GA**: Gestational age; **BW**: Birth weight; **CCC**: Chronic Complex Condition; **COI**: Child Opportunity Index; **LOS:** Length of stay

**Table 2:** Cumulative Cost by Resource Category for Term Newborns Admitted to Intensive Care Units at U.S. Children’s Hospital, 2022–2024. Median [IQR] cumulative cost per patient (*)

Total	Overall	NICU	Non-neonatal ICU
	20,367 [8,336, 59,887]	18,362 [7,934, 50,201]	115,608 [30,151, 259,822]
**Pharmacy**	219 [68, 1,575]	190 [60, 1,072]	5,183 [689, 15,428]
**Imaging**	634 [95, 3,449]	488 [65, 2,727]	8,859 [2,875, 18,890]
**Laboratory**	1,190 [538, 3,434]	1,088 [504, 2,795]	9,349 [2,340, 28,361]
**Clinical**	1,236 [278, 5,395]	1,094 [261, 4,382]	9,127 [1,593, 32,990]
**Supply**	0 [0, 187]	0 [0, 116]	1,048 [0, 7,025]
**Room and Board**	15,180 [6,354, 42,475]	13,802 [6,006, 36,467]	67,078 [18,751, 150,044]

* All values are in U.S. dollars

**Table 3:** Daily Cost by Resource Category for Term Newborns Admitted to Intensive Care Units at U.S. Children’s Hospital, 2022–2024. Median [IQR] daily cost per patient (*)

Total	Overall	NICU	Non-neonatal ICU
	20,367 [8,336, 59,887]	4,293 [2,875, 5,771]	6,796 [5,099, 9,956]
**Pharmacy**	219 [68, 1,575]	49 [16, 148]	337 [145, 631]
**Imaging**	634 [95, 3,449]	108 [12, 321]	538 [301, 928]
**Laboratory**	1,190 [538, 3,434]	245 [126, 450]	641 [381, 1,121]
**Clinical**	1,236 [278, 5,395]	216 [76, 587]	602 [275, 1,289]
**Supply**	0 [0, 187]	0 [0, 19]	65 [0, 256]
**Room and Board**	15,180 [6,354, 42,475]	3,348 [2,185, 4,163]	4,043 [3,449, 5,557]

* All values are in U.S. dollars

Bars represent the median percentage of total hospitalization cost per patient; error bars indicate the interquartile range (P25–P75). Categories are ordered by median cost contribution in the overall cohort.
